# Hierarchical Carbon Architecture Derived from Polybenzoxazine–Melamine Composites: A Strategy for High-Rate Electrochemical Performance

**DOI:** 10.3390/polym18101157

**Published:** 2026-05-08

**Authors:** Thirukumaran Periyasamy, Shakila Parveen Asrafali, Jaewoong Lee

**Affiliations:** Department of Fiber System Engineering, Yeungnam University, Gyeongsan 38541, Republic of Korea; shakilaparveen@yu.ac.kr (S.P.A.); jaewlee@yu.ac.kr (J.L.)

**Keywords:** polybenzoxazine, melamine sponge, carbonization temperature, porous carbon foam, supercapacitor, nitrogen doping, graphitization, electrochemical performance

## Abstract

Developing sustainable, low-cost carbon materials for advanced energy storage remains a major challenge. This study reports the synthesis of three-dimensional (3D) hierarchical porous carbon foams using a polybenzoxazine-coated melamine sponge template, followed by carbonization at 600, 700, and 800 °C. The melamine sponge provided an interconnected porous framework, while nitrogen-rich polybenzoxazine enabled the formation of heteroatom-doped carbon structures. Structural analyses (SEM, XRD, Raman, and XPS) showed that carbonization temperature strongly influenced graphitization, pore development, and nitrogen functionalities. Among all samples, the foam carbonized at 800 °C showed the best electrochemical performance, delivering a specific capacitance of 287 F g^−1^ at 1 A g^−1^, 84% retention at 10 A g^−1^, and 94% capacitance retention after 10,000 cycles. Its superior behavior is attributed to the synergistic effect of improved conductivity, hierarchical porosity, and favorable nitrogen doping. These findings demonstrate that controlled carbonization is an effective strategy for designing high-performance carbon electrodes for supercapacitors, with additional potential in catalysis, adsorption, and EMI shielding.

## 1. Introduction

The global transition toward sustainable energy systems has intensified research efforts focused on developing advanced energy storage devices capable of bridging the gap between power-intensive applications requiring rapid energy delivery and energy-intensive applications demanding high storage capacity. Supercapacitors, also known as electrochemical capacitors or ultracapacitors, occupy a unique position in the energy storage landscape, offering power densities approaching those of conventional capacitors while achieving energy densities intermediate between capacitors and batteries. The superior power capability, exceptional cycling stability, rapid charge–discharge characteristics, and wide operating temperature range of supercapacitors make them indispensable for applications including regenerative braking in electric vehicles, grid-scale energy storage for renewable integration, portable electronics requiring burst power, and backup power systems demanding high reliability [1,2,3,4,5]. However, the relatively modest energy density of supercapacitors compared to batteries represents a fundamental limitation that must be addressed through materials innovation and device engineering to expand their application scope.

The performance of supercapacitors is predominantly determined by the properties of electrode materials, which must simultaneously satisfy multiple demanding requirements including high specific surface area (typically >1000 m^2^ g^−1^) for efficient charge storage, appropriate pore size distribution consisting of micropores (<2 nm) for ion adsorption and mesopores (2–50 nm, ideally 2–10 nm) for rapid electrolyte ion transport, excellent electrical conductivity generally in the range of 10^2^–10^5^ S m^−1^ for carbon-based materials, facilitating electron transfer, chemical stability across wide potential windows, mechanical robustness for long-term cycling, and ideally, pseudocapacitive functionality providing additional charge storage beyond electric double-layer formation. Carbon materials have emerged as the dominant class of supercapacitor electrodes due to their combination of high surface area, good electrical conductivity, excellent chemical stability, wide availability, and relatively low cost [6,7,8]. Various forms of carbon, including activated carbon, carbon nanotubes, graphene, templated mesoporous carbon, and carbon aerogels, have been extensively investigated, each offering distinct advantages and limitations. Activated carbon, despite its high surface area (typically 1000–3000 m^2^ g^−1^) and low cost (commonly US $1–5 kg^−1^ depending on precursor and grade), suffers from poorly defined pore structures, where a large fraction of pores are irregular micropores (<2 nm) that may restrict electrolyte ion accessibility, and limited electrical conductivity, generally in the range of only 10–100 S m^−1^ (significantly lower than graphitic carbons or graphene-based materials). Carbon nanotubes and graphene provide excellent conductivity (typically in the range of 10^4^–10^6^ S m^−1^ for CNT networks and up to 10^6^–10^8^ S m^−1^ for high-quality graphene) and well-defined structures (CNT diameters of 1–50 nm, lengths from micrometers to millimeters, and graphene sheet thickness of approximately 0.34 nm per layer) but face challenges related to aggregation, processing complexity, and high production costs. Three-dimensional carbon foams and aerogels offer hierarchical porous architectures combining micro-, meso-, and macroporosity, facilitating both high surface area and rapid ion transport, while maintaining structural integrity and mechanical compressibility [9,10].

The incorporation of heteroatoms, particularly nitrogen, into carbon frameworks represents a powerful strategy for enhancing supercapacitor performance through multiple mechanisms. Nitrogen doping introduces pseudocapacitive functionality by enabling reversible faradaic reactions at the electrode surface, contributing additional capacitance beyond electric double-layer storage. The presence of nitrogen atoms with different electronegativity compared to carbon creates polarized sites that enhance wettability with aqueous electrolytes, improving ion accessibility to the porous structure. Nitrogen functionalities can improve electrical conductivity by donating electrons to the carbon π-system and provide active sites for electrolyte ion adsorption. The configuration of nitrogen atoms within the carbon lattice, including pyridinic, pyrrolic, graphitic, and oxidized nitrogen species, critically influences the electrochemical properties, with different configurations contributing variably to capacitance, conductivity, and stability. The rational design of nitrogen-doped carbon materials requires control over both the total nitrogen content and the distribution of nitrogen configurations, which can be achieved by selecting appropriate nitrogen-rich precursors and optimizing synthesis conditions, particularly the carbonization temperature [11,12,13,14].

Polybenzoxazine, a class of phenolic resins derived from the ring-opening polymerization of benzoxazine monomers, has attracted increasing attention as a precursor for advanced carbon materials due to several advantageous characteristics. Benzoxazine monomers can be synthesized through a simple one-pot reaction of phenolic compounds, primary amines, and formaldehyde, offering extensive molecular design flexibility through variation in phenolic and amine components. The polymerization of benzoxazines proceeds through a cationic ring-opening mechanism without releasing volatile byproducts, enabling near-zero shrinkage during curing, a significant advantage for producing crack-free carbon structures. Polybenzoxazines exhibit high char yields upon pyrolysis, typically exceeding 60–70%, substantially higher than those of many other polymer precursors, resulting in efficient conversion to carbon materials [15,16,17]. The incorporation of nitrogen atoms from the amine component provides intrinsic nitrogen doping in the resulting carbon materials. The phenolic structure of polybenzoxazine contributes to the formation of graphitic domains upon high-temperature carbonization. The thermal stability and processability of polybenzoxazine enable various fabrication approaches, including coating, casting, and impregnation of porous templates.

Melamine sponge, a three-dimensional open-cell foam composed of melamine-formaldehyde resin, has found widespread use as a cleaning material due to its unique porous architecture, mechanical flexibility, and low cost. The interconnected network of melamine sponge, with characteristic pore sizes ranging from tens to hundreds of micrometers, provides an ideal structural template for producing hierarchical porous carbon materials [18,19,20,21]. The melamine-formaldehyde composition, rich in both nitrogen and carbon, contributes additional heteroatom content to the final carbon product. The commercial availability and scalability of melamine sponge production make it an economically attractive template compared to more expensive alternatives such as silica templates or metal–organic frameworks. The combination of polybenzoxazine coating on melamine sponge templates creates a composite system that leverages the structural advantages of the sponge architecture with the superior carbon-forming properties of polybenzoxazine. The carbonization of this composite system at controlled temperatures enables the production of nitrogen-doped three-dimensional carbon foams with tailored properties for supercapacitor applications [22,23,24].

The carbonization temperature represents one of the most critical parameters influencing the structure and properties of carbon materials derived from polymer precursors. At lower temperatures, typically below 600 °C, the carbonization process primarily involves the removal of volatile species and the initial formation of aromatic structures, resulting in materials with high heteroatom content but limited graphitization and electrical conductivity. At intermediate temperatures in the range of 600–800 °C, progressive graphitization occurs, with increasing alignment and growth of graphitic domains, accompanied by a gradual loss of heteroatoms, particularly oxygen and, to a lesser extent, nitrogen. At higher temperatures, 900 °C, extensive graphitization produces highly ordered carbon structures with excellent electrical conductivity but substantially reduced heteroatom content and potentially decreased surface area due to pore collapse and structural densification. The optimal carbonization temperature for supercapacitor applications must balance competing factors: electrical conductivity, favoring higher temperatures; heteroatom content and surface area, favoring lower temperatures; and mechanical stability, requiring adequate structural integrity. This study systematically investigates the effect of carbonization temperature on polybenzoxazine/melamine sponge-derived carbon foams by comparing materials prepared at three different temperatures (600, 700, and 800 °C), with comprehensive characterization revealing the structural basis for the superior performance of the 800 °C sample.

## 2. Experimental Methodology

### Fabrication of Polybenzoxazine/Melamine Sponge-Based Carbon Foams

**Scheme 1:**
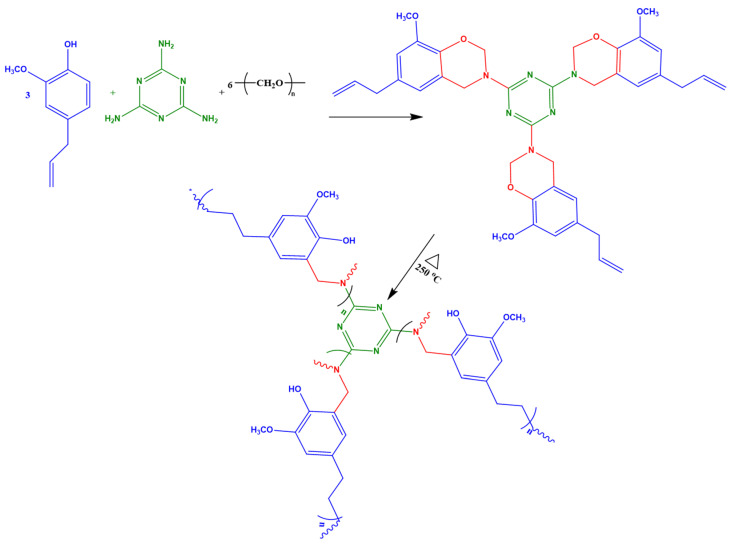
Schematic illustration showing the synthesis of benzoxazine and polybenzoxazine.

A benzoxazine monomer was synthesized from formaldehyde, melamine, and eugenol using the procedure described in our previous work [25] (Scheme 1). Commercial melamine sponge (purchased from CLEANWRAP Co., Ltd., Gangnam-gu, Seoul, Republic of Korea) with a bulk density of approximately 8–10 mg/cm^3^ and an average pore size of 100–200 μm is cut into cubic pieces measuring 2 cm × 2 cm × 2 cm. The melamine sponge pieces are thoroughly cleaned by successive washing with deionized water and ethanol, followed by drying at 60 °C for 12 h to remove any contaminants or residual processing agents. The coating procedure involves immersing the cleaned melamine sponge pieces in a benzoxazine solution prepared by dissolving the synthesized benzoxazine monomer in DMSO at 50 mg/mL. The sponge pieces are allowed to soak in the benzoxazine solution for 2 h with gentle agitation to ensure complete infiltration of the solution into the porous structure. The coated sponges are then removed from the solution and gently compressed to remove excess benzoxazine solution while maintaining uniform coating on the internal surfaces. The coated sponges are dried at room temperature for 12 h, followed by drying at 60 °C for 6 h to remove solvent. The thermal curing of benzoxazine is performed by heating the coated sponges in a convection oven with a programmed temperature profile: ramping from room temperature to 150 °C at 2 °C/min, holding at 150 °C for 2 h, ramping to 180 °C at 2 °C/min, holding at 180 °C for 2 h, and finally ramping to 220 °C at 2 °C/min and holding at 220 °C for 3 h. This multi-step curing protocol ensures complete polymerization of benzoxazine through ring-opening and crosslinking reactions, forming a robust polybenzoxazine coating on the melamine sponge framework.

**Figure 1:**
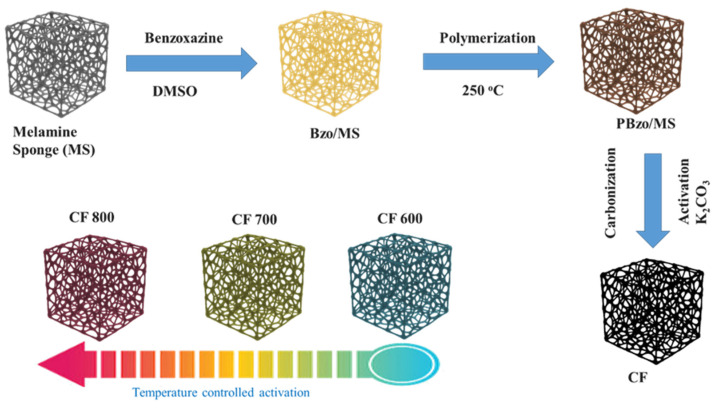
Schematic illustration of the fabrication process of carbon foams (CF) derived from polybenzoxazine/melamine precursors.

The carbonization process is conducted in a tube furnace under a nitrogen atmosphere with a flow rate of 200 mL/min to maintain an inert environment and prevent oxidation. Three separate batches of the polybenzoxazine-coated melamine sponge are carbonized at target temperatures of 600, 700, and 800 °C, respectively. For all samples, the heating protocol involves ramping from room temperature to the target temperature at a rate of 5 °C/min, holding at the target temperature for 2 h to ensure complete carbonization, and then cooling naturally to room temperature under continued nitrogen flow. The resulting carbon foam samples are designated CF 600, CF 700, and CF 800, corresponding to their carbonization temperatures, as depicted in Figure 1. The prepared samples maintain the three-dimensional macroscopic structure of the original melamine sponge, but with significantly reduced dimensions due to shrinkage during carbonization, typically retaining approximately 75–80% of the original linear dimensions. The mass yield of carbon foam relative to the initial mass of polybenzoxazine-coated melamine sponge is approximately 28–35%, with higher yields observed at lower carbonization temperatures due to greater retention of heteroatoms and less extensive decomposition.

## 3. Results and Discussion

### 3.1. Structural Characterization and Temperature-Dependent Evolution

**Figure 2:**
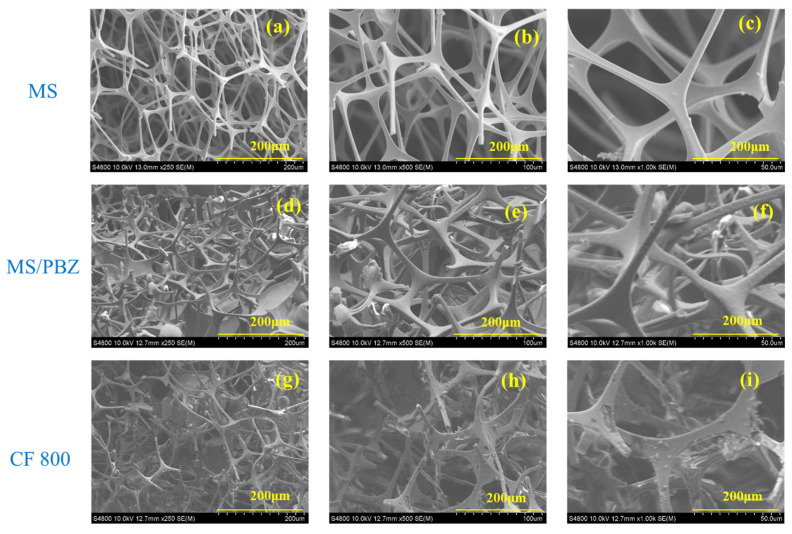
SEM images of (**a**–**c**) MS, (**d**–**f**) MS/PBZ, (**g**–**i**) CF 800.

**Figure 3:**
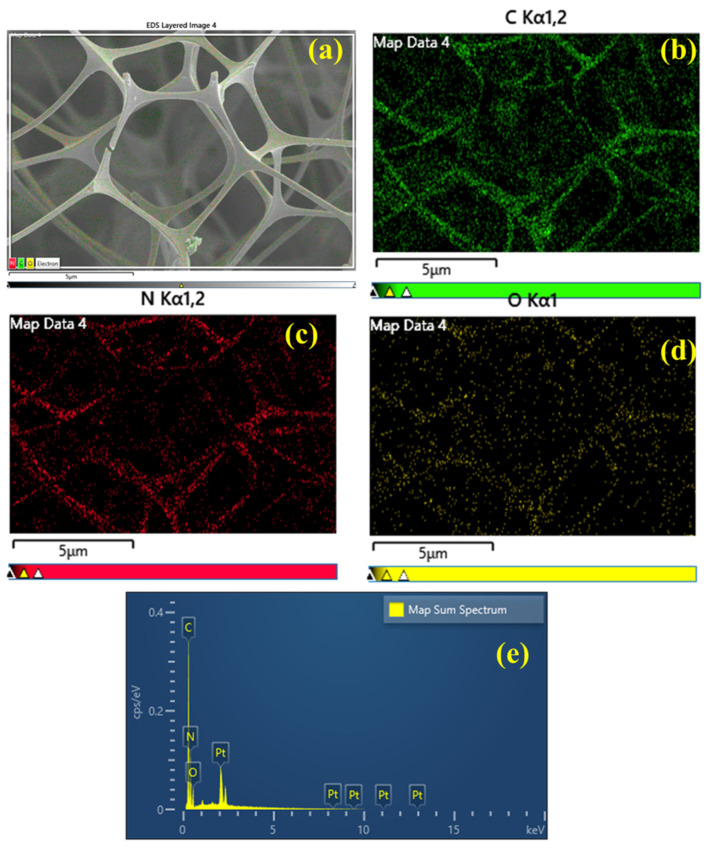
(**a**–**d**) SEM elemental mapping images and (**e**) SEM-EDS analysis of CF 800.

**Figure 4:**
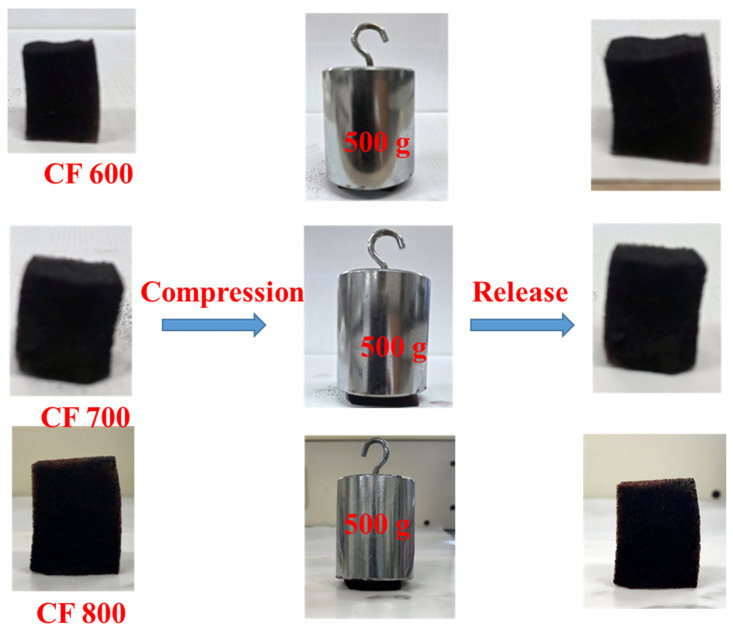
Digital photographs displaying the compressibility of CF 600, CF 700, and CF 800.

The preliminary analysis confirming the structure of the synthesized benzoxazine monomer was performed using FT-IR, ^1^H-NMR, and ^13^C-NMR, and reported in our previous work [25]. The reference paper contains the details of the FTIR results. The explanation could be found under Section 3.1 of the Results and Discussion. By using FT-IR, ^1^H, and ^13^C-NMR techniques, a characterization of the prepared benzoxazine monomer was conducted before curing. The condensation of eugenol, melamine, and formaldehyde was used to synthesize EM-Bzo (Figure 1a). In the FT-IR spectrum (Figure 1b), EM-Bzo exhibits a characteristic peak at 939 cm^−1^, denoting oxazine ring structure at 1246 and 1022 cm^−1^ due to the asymmetric and symmetric vibration of C–O–C, respectively. A band can be seen at 1142 cm^−1^ for the C–N–C stretching vibrations, as well as a peak at 1372 cm^−1^ caused by tetra-substituted benzene [24]. Furthermore, methoxy carbonyl bound to the benzene ring exhibited symmetric and asymmetric vibrations at 1021 and 1247 cm^−1^, respectively. Moreover, eugenol’s alkyl chain, as well as the methylene group of its benzoxazine ring, gave bands between 2976 and 2828 cm^−1^, respectively [25]. Morphological characterization by scanning electron microscopy reveals that all three carbon foam samples maintain the three-dimensional interconnected network structure of the original melamine sponge template, demonstrating the effectiveness of the polybenzoxazine coating and carbonization strategy in preserving the macroscopic architecture [26,27,28]. The CF 600 sample exhibits a relatively smooth surface texture on individual struts with limited visible porosity at high magnification, suggesting incomplete graphitization and the presence of substantial amorphous carbon regions. The strut diameter in CF 600 ranges from approximately 8 to 15 μm, with the polybenzoxazine-derived carbon forming a continuous coating on the underlying melamine-derived carbon framework. The interconnected macropores between struts maintain dimensions of 50–150 μm, providing open channels for electrolyte penetration and ion transport. The CF 700 sample displays notable changes in surface morphology compared to CF 600, with increased surface roughness and the emergence of small pores and cracks on strut surfaces. These features indicate progressive decomposition of less stable carbon structures and the beginning of graphitic domain formation and alignment. The strut diameter in CF 700 shows a slight reduction to 7–13 μm due to continued densification and the removal of volatile species during carbonization at higher temperatures. The macroporous structure remains well-preserved, with pore sizes similar to those of CF 600, confirming the structural stability of the carbon foam architecture at this intermediate temperature. The CF 800 sample exhibits the most developed hierarchical porous structure with distinctly rough strut surfaces featuring abundant micropores and mesopores visible in high-magnification SEM images (Figure 2 and Figure 3) (Table 1). The strut diameter further decreases to approximately 6–12 μm, reflecting the most extensive densification and structural optimization. Importantly, the macroporous network structure remains intact without collapse or severe deformation, demonstrating that the 800 °C carbonization temperature achieves an optimal balance between graphitization-induced densification and structural integrity preservation (Figure 4). The hierarchical porous architecture, combining macropores for rapid electrolyte access, mesopores for ion transport, and micropores for charge storage, represents an ideal configuration for supercapacitor electrodes, explaining in part the superior performance of CF 800 [29,30,31,32,33].

**Figure 5:**
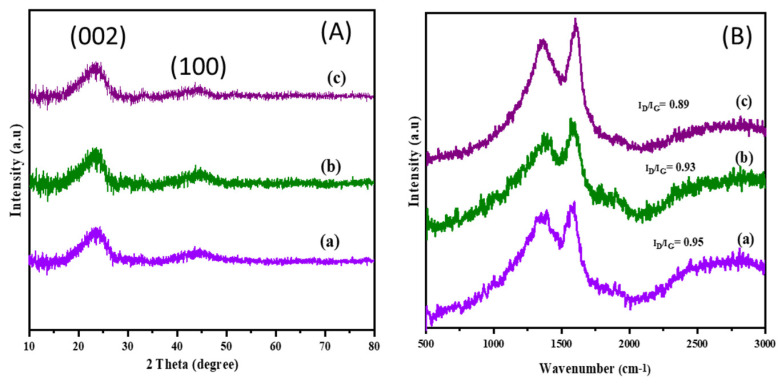
(**A**) XRD patterns and (**B**) Raman spectra of (a) CF 600, (b) CF 700, and (c) CF 800.

X-ray diffraction patterns of the three carbon foam samples provide complementary information about crystalline structure and graphitization degree. All three samples display two characteristic broad peaks: a (002) peak around 2θ = 24–26° corresponding to the interlayer spacing of graphitic carbon, and a (100) peak around 2θ = 43° corresponding to the in-plane lattice parameter (Figure 5A). The CF 600 sample shows the broadest and least intense (002) peak centered at 2θ = 24.2°, indicating large interlayer spacing (d_002_ = 0.367 nm calculated using Bragg’s law) and small crystallite size in the c-axis direction (L_c_ = 1.8 nm calculated using the Scherrer equation). These parameters confirm the predominantly amorphous nature of CF 600 with limited graphitic ordering. The CF 700 sample exhibits a sharper and more intense (002) peak shifted to 2θ = 25.1°, corresponding to a reduced interlayer spacing (d_002_ = 0.355 nm) and increased crystallite size (L_c_ = 2.6 nm), indicating enhanced graphitization compared to CF 600. The CF 800 sample displays the sharpest and most intense (002) peak centered at 2θ = 25.8°, yielding d_002_ = 0.345 nm and L_c_ = 3.4 nm, approaching the values of well-graphitized carbon materials. The progressive shift in the (002) peak to higher 2θ angles and the corresponding decrease in interlayer spacing with increasing carbonization temperature reflect the removal of defects and heteroatoms, allowing closer packing of graphitic layers and improved structural ordering [34,35].

Raman spectroscopy provides sensitive detection of carbon structural features and defects through characteristic vibrational modes. The Raman spectra of all three carbon foam samples display the characteristic D band around 1345 cm^−1^ and G band around 1580 cm^−1^, with the relative intensities of these bands varying with carbonization temperature (Figure 5B). The CF 600 sample exhibits an I_D_/I_G_ ratio of 1.08, indicating high defect density and limited graphitization consistent with the XRD observations. The CF 700 sample shows a slightly decreased I_D_/I_G_ ratio of 1.02, suggesting a modest reduction in defect density and improved graphitic ordering. Interestingly, the CF 800 sample displays an I_D_/I_G_ ratio of 0.95, the lowest among the three samples, confirming the highest degree of graphitization and the most ordered carbon structure [36,37,38]. The 2D band around 2690 cm^−1^ becomes more prominent in the CF 800 sample compared to CF 600 and CF 700, further supporting enhanced graphitic layer stacking. The full width at half maximum (FWHM) of the G band decreases progressively from CF 600 (92 cm^−1^) to CF 700 (78 cm^−1^) to CF 800 (68 cm^−1^), indicating increasing crystalline perfection and reduced structural heterogeneity at higher carbonization temperatures. These Raman spectroscopy results provide strong evidence for the superior structural quality of CF 800, which directly translates to enhanced electrical conductivity and electrochemical performance.

**Figure 6:**
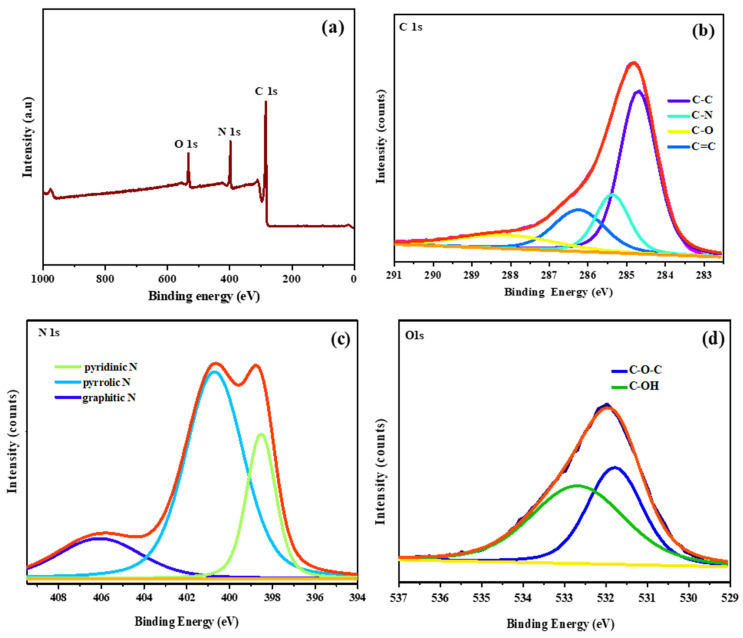
XPS spectrum showing the (**a**) survey spectrum and (**b**–**d**) deconvoluted spectrum of CF 800.

X-ray photoelectron spectroscopy analysis provides critical information about surface elemental composition and the chemical states of heteroatoms, particularly nitrogen, which plays a crucial role in pseudocapacitive performance. The survey XPS spectrum of CF 800 (Figure 6) shows prominent peaks corresponding to carbon, nitrogen, and oxygen, and their energies are tabulated in Table 2 and Table 3. The high-resolution N 1s-deconvoluted XPS spectrum reveals four nitrogen species with distinct binding energies and electrochemical functionalities (Figure 6c). Pyridinic-N (398.5 eV) refers to nitrogen atoms bonded to two carbon atoms at the edges of graphene layers or in six-membered rings, contributing to pseudocapacitance through reversible redox reactions and enhancing wettability. Pyrrolic-N (401.0 eV) corresponds to nitrogen atoms in five-membered heterocyclic rings and also contributes to pseudocapacitance, improving electrical conductivity through electron donation. Graphitic-N (405.0 eV) corresponds to nitrogen atoms substituting for carbon atoms within the graphene lattice, thereby enhancing electrical conductivity and providing catalytic activity. The relative proportions of these nitrogen species change significantly with carbonization temperature. The high proportion of graphitic-N in CF 800, combined with substantial retention of pyridinic-N and pyrrolic-N, creates an optimal nitrogen configuration that balances conductivity enhancement from graphitic-N with pseudocapacitive contributions from pyridinic-N and pyrrolic-N, explaining the superior electrochemical performance despite lower total nitrogen content [39,40,41,42,43].

### 3.2. Electrochemical Performance Evaluation

**Figure 7:**
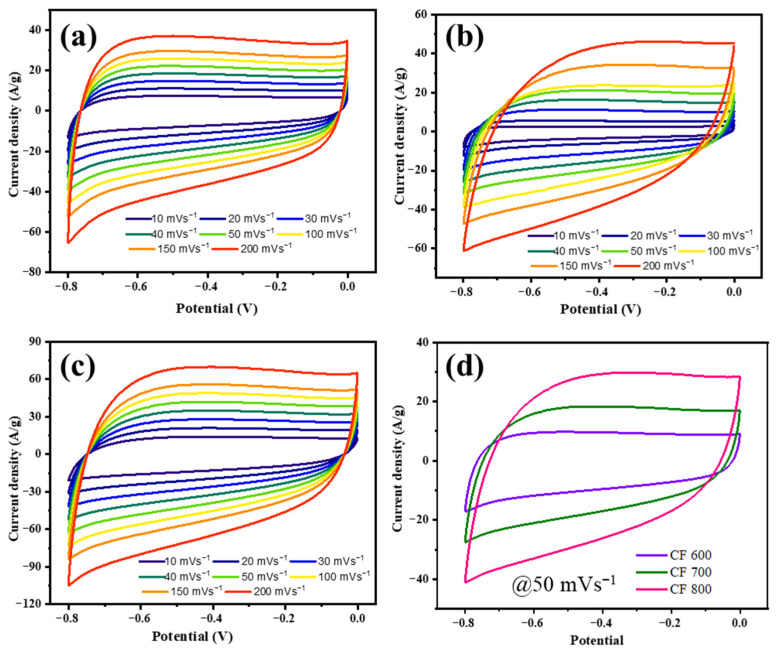
Cyclic voltammetry (CV) curves of (**a**) CF 600, (**b**) CF 700, and (**c**) CF 800 at scan rates ranging from 10 to 200 mV s^−1^. (**d**) Comparison of CV curves at 50 mV s^−1^.

The electrochemical measurements were carried out in a three-electrode configuration using 1 M KOH aqueous solution as the electrolyte, with the prepared carbon foam serving as the working electrode, a platinum wire as the counter electrode, and an Ag/AgCl electrode as the reference. The working electrode was fabricated by mixing the active material with carbon black and PVDF binder in a weight ratio of 80:10:10 using NMP as the solvent to form a homogeneous slurry, which was then coated onto the substrate and dried at 60–80 °C, with an active material loading of approximately 8 mg. Cyclic voltammetry was conducted at scan rates ranging from 5 to 100 mV s^−1^, while galvanostatic charge–discharge measurements were performed at current densities between 0.5 and 10 A g^−1^. Electrochemical impedance spectroscopy was carried out over a frequency range of 100 kHz to 0.01 Hz with an amplitude of 5 mV, and all measurements were conducted at room temperature. The electrochemical characterization through cyclic voltammetry reveals distinct differences in the capacitive behavior of the three carbon foam samples. The CV curves of CF 600 at various scan rates display quasi-rectangular shapes characteristic of electric double-layer capacitance, with slight distortions indicating limited pseudocapacitive contributions. The enclosed area of the CV curves, proportional to the stored charge, is moderate, and the curves show significant distortion at high scan rates (10–200 mV/s), indicating poor rate capability due to limited electrical conductivity and restricted ion transport in the predominantly microporous structure (Figure 7). The CF 700 sample exhibits improved CV curve shapes with larger enclosed areas and better maintenance of rectangular profiles at high scan rates compared to CF 600, demonstrating enhanced capacitive performance. However, the improvement is modest, suggesting that carbonization at 700 °C, while beneficial, does not fully optimize the material properties. The CF 800 sample displays the most impressive CV characteristics with the largest enclosed areas across all scan rates, indicating the highest capacitance. The CV curves maintain excellent rectangular shapes even at very high scan rates of 200 mV/s, demonstrating outstanding rate capability. Notably, the CV curves of CF 800 show subtle redox peaks or humps, particularly in the potential region of −0.8 to −0.6 V vs. SCE, indicating significant pseudocapacitive contributions from nitrogen functional groups [44,45]. These pseudocapacitive features are most pronounced in CF 800 despite its lower total nitrogen content compared to CF 600 and CF 700, confirming that the favorable nitrogen configuration (high graphitic-N content while retaining active pyridinic-N and pyrrolic-N) in CF 800 provides more effective pseudocapacitive functionality.

**Figure 8:**
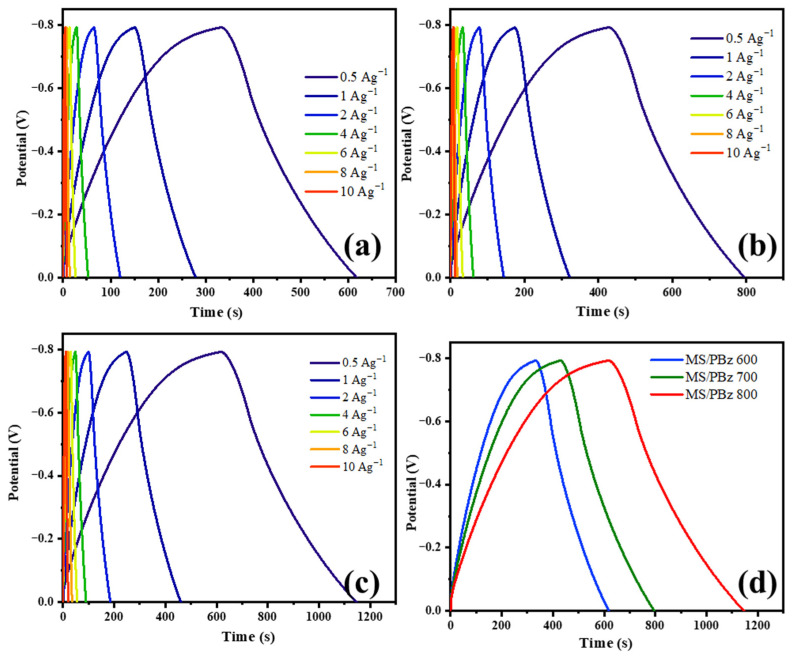
GCD curves of (**a**) CF 600, (**b**) CF 700, and (**c**) CF 800 at 0.5 to 10 A/g, and (**d**) comparison of GCD curves at 0.5 A/g.

Galvanostatic charge–discharge measurements provide quantitative assessments of specific capacitance and rate capability. The GCD curves of all three samples display characteristic triangular shapes with slight curvature, consistent with the combined contributions of electric double-layer capacitance and pseudocapacitance (Figure 8). The CF 600 sample exhibits a specific capacitance of 193 F/g @ 0.5 A/g, which decreases to 174 F/g @ 1 A/g and 51 F/g @ 10 A/g, representing capacitance retentions of 87% and 26% at these high current densities. The poor rate capability of CF 600 is attributed to its limited electrical conductivity, a predominantly microporous structure that restricts rapid ion transport, and an unfavorable nitrogen configuration. The CF 700 sample shows improved performance with specific capacitance of 249 F/g @ 0.5 A/g, decreasing to 203 F/g @ 1 A/g and 81 F/g @ 20 A/g, corresponding to capacitance retentions of 81% and 33%. The enhanced performance of CF 700 compared to CF 600 reflects the improved graphitization, increased mesoporosity, and more favorable nitrogen configuration achieved at the higher carbonization temperature [46,47,48]. The CF 800 sample demonstrates exceptional performance with a specific capacitance of 359 F/g @ 0.5 A/g, the highest among the three samples and representing 46% and 31% improvements over CF 600 and CF 700, respectively. More impressively, CF 800 maintains specific capacitances of 279 F/g @ 1 A/g and 125 F/g @ 10 A/g, corresponding to outstanding capacitance retentions of 85% and 78%. The superior rate capability of CF 800 is attributed to the synergistic combination of excellent electrical conductivity from optimal graphitization, a hierarchical porous structure that facilitates rapid ion transport, a high surface area that maximizes active sites, and a favorable nitrogen-doping configuration that provides both conductivity enhancement and pseudocapacitive contributions. The discharge time of CF 800 @ 1 A/g reaches 230 s, substantially longer than CF 700 (203 s) and CF 600 (174 s), directly reflecting the higher energy storage capacity. The IR drop at the beginning of discharge, which indicates the equivalent series resistance, is smallest for CF 800 (approximately 0.015 V), compared to 0.028 V for CF 700 and 0.045 V for CF 600, confirming the superior electrical conductivity of the 800 °C carbonized sample.

**Figure 9:**
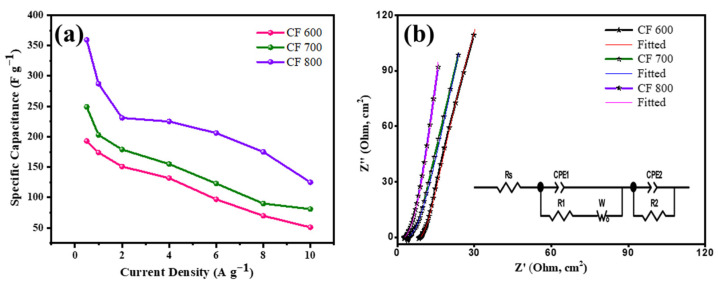
Comparison of the electrochemical performance of CF 600, CF 700, and CF 800: (**a**) specific capacitances at different current densities and (**b**) Nyquist plots.

Electrochemical impedance spectroscopy provides detailed information on resistance and charge-transport characteristics. The Nyquist plots of all three samples display a semicircle in the high-frequency region corresponding to charge transfer resistance, followed by a straight line in the low-frequency region corresponding to ion diffusion (Figure 9). The CF 600 sample exhibits the largest semicircle diameter, approximately 3.1 Ω, indicating substantial charge-transfer resistance that limits electrochemical performance. The intercept with the real axis at high frequency, representing equivalent series resistance, is 0.74 Ω for CF 600. The slope of the low-frequency straight line is relatively low, indicating slow ion diffusion kinetics. The CF 700 sample shows reduced charge transfer resistance, with a semicircle diameter of approximately 2.3 Ω and an equivalent series resistance of 0.53 Ω, demonstrating improved charge transport compared to CF 600. The CF 800 sample displays the most favorable impedance characteristics with the smallest semicircle diameter of approximately 1.2 Ω and the lowest equivalent series resistance of 0.36 Ω, confirming excellent charge transfer and minimal resistive losses [49,50,51,52,53]. The low-frequency straight line of CF 800 exhibits the steepest slope approaching 90°, indicating ideal capacitive behavior with rapid ion diffusion. The characteristic frequency, defined as the frequency at which the imaginary impedance reaches maximum in the semicircle region, is highest for CF 800 (12.5 Hz) compared to CF 700 (6.8 Hz) and CF 600 (3.2 Hz), indicating the fastest charge–discharge kinetics. The time constant, calculated as the reciprocal of characteristic frequency, is shortest for CF 800 (0.08 s), demonstrating its superior power capability. These EIS results provide strong evidence that the 800 °C carbonization temperature optimizes the electrical and ionic conductivity of the carbon foam, enabling rapid charge–discharge and minimizing energy losses during operation.

**Figure 10:**
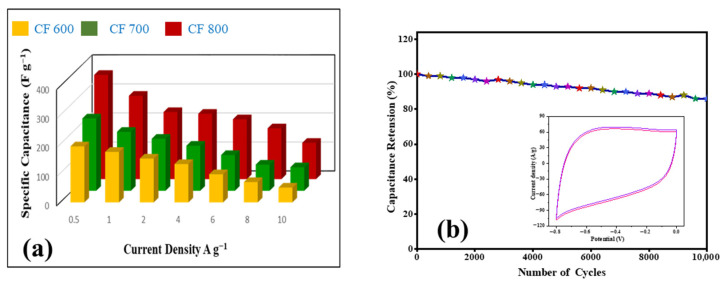
(**a**) Comparison of the electrochemical performance of CF 600, CF 700, and CF 800 (**b**) Cycling stability of CF 800.

**Figure 11:**
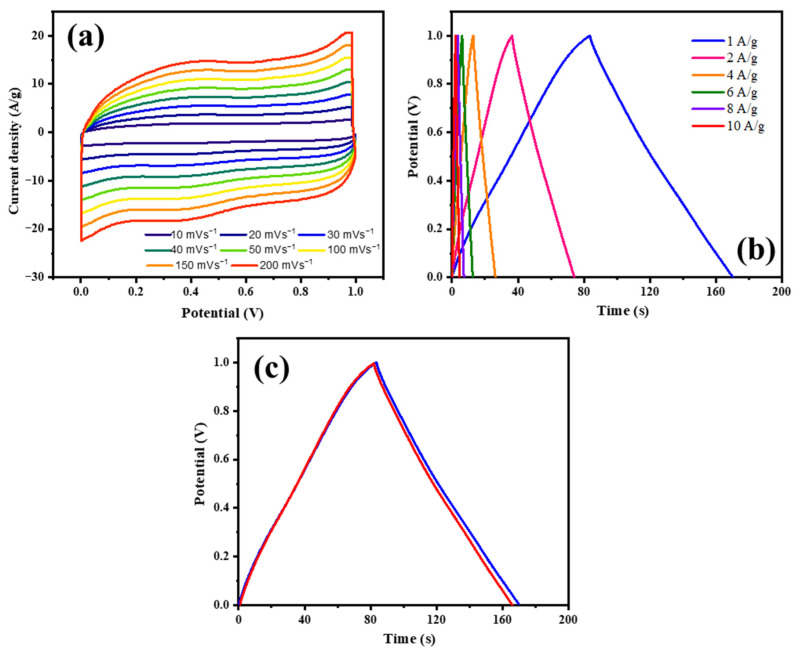
Electrochemical performance of CF 800-based symmetric supercapacitors (CF 800//CF 800): (**a**) CV curves, (**b**) galvanostatic charge–discharge (GCD) profiles, and (**c**) GCD profiles at 1 A g^−1^ measured at the first cycle and after 5000 cycles.

The cycling stability evaluation through 10,000 continuous charge–discharge cycles reveals the long-term durability of the carbon foam electrodes (Figure 10). The CF 800 sample demonstrates outstanding cycling stability, maintaining 86% of its initial capacitance after 10,000 cycles, among the best reported for nitrogen-doped carbon materials. The exceptional stability of CF 800 is attributed to its robust three-dimensional network structure, optimal graphitization, which provides chemical stability and mechanical strength, and the favorable nitrogen configuration, which resists degradation. Post-cycling SEM examination of CF 800 electrodes reveals that the hierarchical porous structure remains intact, without collapse or severe damage, confirming the structural resilience that underlies the excellent cycling stability. A symmetric two-electrode system was constructed using CF 800 [CF 800//CF 800] and its performance is given in Figure 11, showing specific capacitance of 72 F/g @ 1 A/g. Notably, the symmetric electrode, CF 800//CF 800, has a lower specific capacitance than its representative three-electrode system. This is because, in a three-electrode system, the measured capacitance mainly reflects the performance of the single working electrode. In contrast, in a symmetric two-electrode system, both electrodes contribute to the capacitance. For two identical electrodes, the total device capacitance is determined by two electrodes in series, i.e., 1/C_cell_ = 1/C_1_ + 1/C_2_. Therefore, C_cell_ = C/2, and the device capacitance is lower. The higher specific capacitance observed in the three-electrode configuration arises from isolated testing of the working electrode under ideal potential control with minimized internal resistance. In contrast, the symmetric two-electrode device reflects the realistic full-cell behavior where two electrodes act in series, leading to reduced overall capacitance [54,55,56,57].

### 3.3. Comparison with the Literature and Performance Benchmarking

The electrochemical performance of the CF 800 sample compares favorably with other nitrogen-doped carbon materials reported in the literature for supercapacitor applications (Table 4). Activated carbon-based electrodes typically achieve specific capacitances in the range of 150–250 F/g in aqueous electrolytes, with rate capability often limited by poorly defined pore structures and low electrical conductivity. Graphene-based materials can achieve higher capacitances of 200–300 F/g with better rate capability, but face challenges related to restacking, processing complexity, and high cost. Carbon nanotube-based electrodes offer excellent conductivity and defined structures but typically show specific capacitances of 100–200 F/g due to their limited surface area [38,39,40,41,42,43]. Heteroatom-doped carbon materials derived from various precursors, including biomass, polymers, and metal–organic frameworks, have been extensively studied, with nitrogen-doped carbons generally showing superior performance due to pseudocapacitive contributions.

The CF 800 sample’s specific capacitance of 359 F/g @ 0.5 A/g ranks among the highest reported for nitrogen-doped porous carbons in three-electrode configurations with aqueous electrolytes. More importantly, the rate capability with 86% capacitance retention @ 5 A/g surpasses most reported carbon materials, which typically show capacitance retentions of 50–70% at comparable current densities. The cycling stability of 86% retention after 10,000 cycles is exceptional, with many nitrogen-doped carbons showing 80–90% retention under similar testing conditions. The combination of high capacitance, excellent rate capability, and outstanding cycling stability in CF 800 demonstrates the effectiveness of the polybenzoxazine/melamine sponge approach with optimized carbonization at 800 °C. The scalability and relatively low cost of the precursor materials and synthesis process represent additional advantages compared to more exotic carbon materials requiring complex synthesis or expensive precursors.

### 3.4. Mechanistic Understanding of Temperature-Dependent Performance

The superior performance of the CF 800 sample compared to CF 600 and CF 700 can be understood by considering multiple interconnected factors. The degree of graphitization, as evidenced by XRD and Raman analyses, is optimal in CF 800, providing excellent electrical conductivity that minimizes resistive losses and enables rapid electron transfer. The electrical conductivity, while not directly measured in this study, can be inferred from the low equivalent series resistance and small IR drop in electrochemical measurements, with CF 800 clearly exhibiting the lowest resistance. The hierarchical porous structure in CF 800, combining microporosity for high surface area, mesoporosity for ion transport, and macroporosity for an electrolyte reservoir, creates an ideal architecture that maximizes both charge storage capacity and accessibility. In CF 800, abundant active sites for electric double-layer formation directly contribute to high capacitance.

The carbonization temperature of 800 °C represents an optimal point where competing processes balance favorably. At lower temperatures (600 °C), insufficient graphitization results in poor electrical conductivity despite high heteroatom content and surface area. At intermediate temperatures (700 °C), graphitization improves but remains incomplete, and the porous structure is not fully developed. At 800 °C, graphitization reaches an optimal level, providing excellent conductivity while avoiding excessive graphitization that would reduce heteroatom content and surface area. The porous structure is fully developed by removing unstable carbon and heteroatom groups, creating hierarchical porosity without structural collapse. The nitrogen configuration evolves to the most favorable distribution with high graphitic-N for conductivity and retained pyridinic-N and pyrrolic-N for pseudocapacitance. Higher carbonization temperatures above 800 °C, while potentially increasing graphitization further, would likely result in excessive nitrogen loss, reduced surface area through pore collapse, and diminished pseudocapacitive functionality, ultimately decreasing overall capacitive performance [48,55].

## 4. Conclusions and Future Perspectives

This comprehensive study demonstrates that the carbonization temperature critically influences the structure and electrochemical performance of polybenzoxazine/melamine sponge-derived carbon foams for supercapacitor applications. Through systematic comparison of samples carbonized at 600, 700, and 800 °C, we establish that the 800 °C sample exhibits superior performance across all evaluated metrics, including specific capacitance, rate capability, cycling stability, and mechanical compressibility. The exceptional performance of CF 800 is attributed to the synergistic combination of optimal graphitization providing excellent electrical conductivity, hierarchical porous structure with surface area of 1245 m^2^/g facilitating both high charge storage and rapid ion transport, and favorable nitrogen doping configuration with high graphitic-N content for conductivity enhancement and retained pyridinic-N and pyrrolic-N for pseudocapacitive contributions.

The polybenzoxazine/melamine sponge approach represents a scalable and cost-effective strategy for producing high-performance nitrogen-doped carbon materials, leveraging the structural advantages of commercial melamine sponge templates with the superior carbon-forming properties of polybenzoxazine. The utilization of readily available precursor materials and straightforward synthesis procedures positions this approach as potentially viable for industrial-scale production, addressing the critical need for economically feasible advanced electrode materials for energy storage devices. The three-dimensional interconnected network structure with excellent mechanical compressibility opens possibilities for applications beyond conventional supercapacitors, including flexible and wearable energy storage devices, compressible electrodes for pressure-responsive systems, and structural energy storage materials.

Future research directions should focus on several key areas to further advance this technology. The optimization of polybenzoxazine coating thickness and uniformity could enhance performance by increasing active material loading while maintaining the advantageous porous structure. The exploration of different benzoxazine monomer structures with varied phenolic and amine components could enable tuning of nitrogen content, carbon yield, and functional properties. The investigation of carbonization temperatures beyond 800 °C, potentially up to 900–1000 °C, could determine whether further performance improvements are possible or whether 800 °C represents a true optimum. The development of two-electrode symmetric supercapacitor devices using CF 800 as both positive and negative electrodes would enable practical performance evaluation in device configurations relevant for applications. The exploration of organic electrolytes or ionic liquids to expand the operating voltage window could significantly increase energy density while maintaining the excellent rate capability and cycling stability demonstrated in aqueous electrolytes. Investigating additional functional modifications, such as the incorporation of metal oxides or conducting polymers, could yield hybrid materials with further enhanced performance. A comprehensive techno-economic analysis and life-cycle assessment would provide crucial information on the commercial viability and environmental sustainability of the polybenzoxazine/melamine sponge approach compared to competing technologies.

## Figures and Tables

**Table 1:** Elemental composition of CF 800 obtained from SEM-EDS analysis.

Element	Line	Apparent Concentration	Intensity Correction	k Ratio	Wt%	Atomic %
C	K series	10.4	0.35	0.1043	38.0	42.3
N	K series	21.1	0.55	0.0376	48.8	46.7
O	K series	2.3	0.22	0.0077	13.2	11.0

**Table 2:** XPS data of CF 800 showing quantitative analysis of elements.

Element	Start BE	FWHM eV	Atomic %
C 1s	297.98	2.15	39.5
N 1s	409.98	1.43	47.3
O 1s	544.98	1.83	13.2

**Table 3:** XPS data of CF 800 showing observed transitions and their energies.

Element	Observed Transitions	Energies (eV)
C 1s	C–C	284.6
	C–N	285.3
	C–O	288.2
	C=C	286.5
N 1s	Pyridinic N	398.5
	Pyrrolic N	401.0
	Graphitic N	405.0
O 1s	C–O–C	532.0
	C–OH	533.0

**Table 4:** Comparison of recently reported nitrogen-doped carbon materials derived from similar precursors.

Precursor	Structure	SSA (m^2^/g)	N Content (%)	Capacitance (F/g)	Rate Retention (%)	Cycling Stability	Ref.
Melamine sponge	3D foam	~800–1200	5–10	220–300 @ 1 A/g	60–75	~85% (5 k cycles)	[18,19]
Polybenzoxazine	Porous carbon	~600–900	4–8	250–320 @ 1 A/g	65–80	~90% (5 k cycles)	[29,30]
Polydopamine	Nanospheres	~500–700	6–10	200–280 @ 1 A/g	60–70	~85%	[23]
Lignin/melamine	Hierarchical	~1000–1500	3–6	250–340 @ 1 A/g	70–80	~90%	[48,49]
Melamine + CNT	Hybrid	—	—	150–250	High	Good	[12]
PBZ + melamine sponge	3D hierarchical foam	1245	~3–5	359 @ 0.5 A/g	~78% @ 10 A/g	94% (10 k cycles)	This work (CF 800)

## Data Availability

The original contributions presented in this study are included in the article. Further inquiries can be directed to the corresponding author.
